# Energy calibration of the 2.5 MV Pelletron at the Dalton Cumbrian Facility

**DOI:** 10.1140/epja/s10050-025-01622-5

**Published:** 2025-07-08

**Authors:** K. Linkowski, R. S. Sidhu, J. Skowronski, M. Aliotta, P. Black, T. Davinson, M. Wiescher, A. Caciolli, J. Jones, K. Manukyan, D. Robertson, A. Smith

**Affiliations:** 1 School of Physics and Astronomy, The University of Edinburgh, EH9 3FD Edinburgh, UK; 2 School of Mathematics and Physics, University of Surrey, GU2 7XH Guildford, UK; 3 Università Degli Studi di Padova, 35131 Padova, Italy; 4 INFN, Sezione di Padova, 35131 Padova, Italy; 5 Department of Physics and Astronomy, University of Notre Dame, 46556 Notre Dame, IN USA; 6 The University of Manchester’s Dalton Cumbrian Facility, Westlakes Science Park, Moor Row, Cumbria, CA24 3HA UK

## Abstract

We report on the energy calibration of the 2.5 MV Pelletron accelerator at the Dalton Cumbrian Facility in England (UK) using five well-known resonances in the ^27^Al(*p*,$\gamma )^{28}$Si reaction in the proton beam energy range of 632–1800 keV. The beam energy spread was also measured and found to be 191(38) eV. Additionally, we checked the stability and reproducibility of the accelerator’s beam energy, confirming its suitability for nuclear astrophysics experiments, especially in the high-energy regime of stellar evolution and nucleosynthesis.

**Supplementary Information:**

The online version contains supplementary material available at 10.1140/epja/s10050-025-01622-5.

## Introduction

Nuclear reactions of astrophysical interest can be studied in laboratory settings, where particle accelerators deliver high-intensity ion beams – often protons or alpha particles – onto targets that contain the desired reactant nuclei [1, 2]. However, studying these reactions at (sub-Coulomb) astrophysical energies poses challenges due to the exponential drop in the reaction cross section associated with the Coulomb barrier between the interacting nuclei [3, 4]. This means that a precise knowledge of the interaction energy is critical for the accurate evaluation of cross-section data at low energies, because even small uncertainties in the interaction energy can lead to significant uncertainties in the reaction cross-section. As an example, in the ^14^N($p,\gamma )^{15}$O reaction, the slowest reaction in the CNO cycle, an uncertainty of 1.5 keV in the proton beam energy at $E_p$ = 100 keV translates into a 20% change in the cross-section [5, 6].

**Fig. 1:**
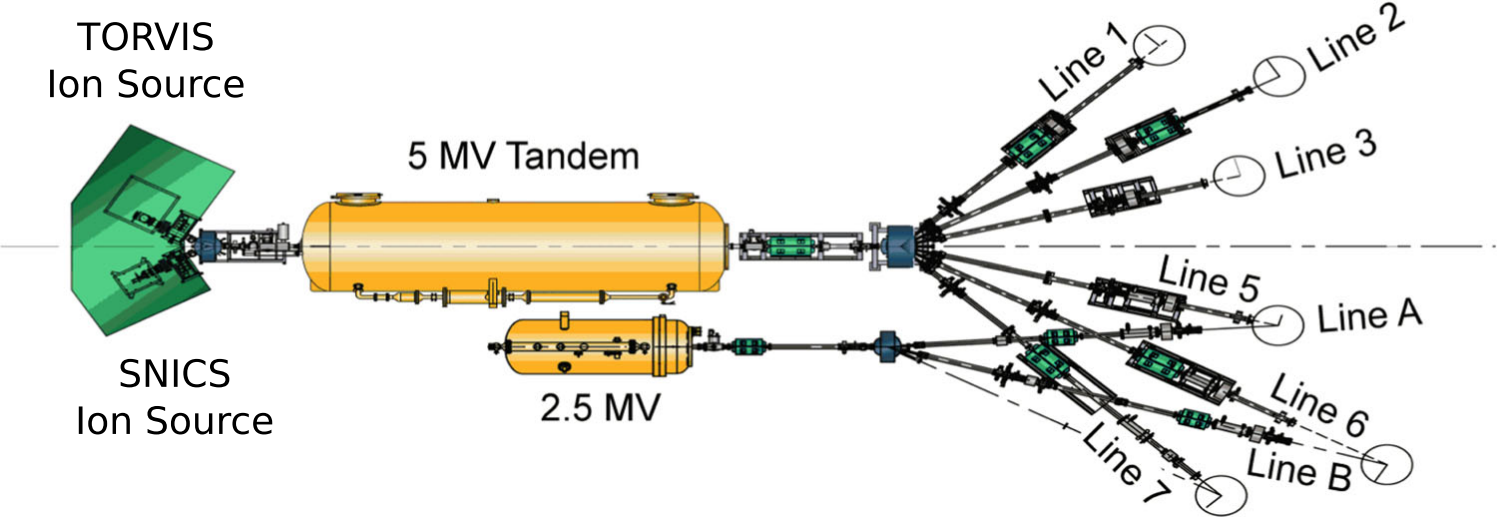
Schematic view of the accelerator complex at the Dalton Cumbrian Facility (UK), adapted from [9]. The present study reports the energy calibration of the 2.5 MV Pelletron using a setup installed on Line A (see text for details)

In this paper, we assess the suitability of the Dalton Cumbrian Facility (DCF) [7] in Cumbria, England, for nuclear astrophysics studies. Managed by the University of Manchester, the facility has been built to allow academia and industry the opportunity to carry out research in radiation science and nuclear engineering decommissioning [8, 9]. DCF houses two accelerators, a 2.5 MV single-ended Pelletron, and a 5 MV tandem, arranged in a system that allows for a range of ion irradiation and analysis capabilities across eight beam lines (see Fig. 1). Here, we focus on the beam energy calibration of the 2.5 MV Pelletron accelerator only.

The energy calibration of a particle accelerator can be achieved by relating the accelerator’s settings and voltage parameters to well-known energy references. The standard approach exploits thick-target yields [10] of known resonances in proton induced radiative capture reactions, measured as a function of beam energy in small energy steps (typically $\le 0.3$ keV). The thick-target method is chosen because the midpoint of the steep rise of the leading edge in the yield curve corresponds to the known resonance energy in the laboratory reference frame. Thus, the accelerator parameters required to reach the midpoint can easily be related to the known energy and a calibration curve can be attained. For the thick-target method to be applicable, the natural resonance width ($\varGamma$) must be much smaller than the energy lost by the beam in traversing the target in the center-of-mass frame, i.e. $\varGamma \ll \varDelta E_{\mathrm{c.m.}}$ [11].

For the present study, we used five well-known narrow resonances in the ^27^Al($p,\gamma )^{28}$Si reaction, with energies and widths reported in Table 1. However, rather than changing the proton beam energy by slowly varying the accelerator settings, which would be very time consuming, a different approach was used instead. Namely, initial accelerator settings, i.e. Terminal Voltage (TV) and Extraction Voltage (EV), were tentatively chosen to achieve a nominal energy higher than that of the resonance energy. A positive bias voltage was then applied to the ^27^Al target via a power supply so as to effectively slow down the proton-beam energy in small increments until a full thick-target scan around the resonance energy could be performed. This approach allowed us to change the beam energy on target in fine steps of 0.1 keV (see Sect. 3).

**Table 1:** Resonance energies and widths for the five narrow resonances in the ^27^Al($p,\gamma )^{28}$Si reaction used in this study. Values are taken from Reference [12]

Resonance energy	Resonance width
$E_{\mathrm{{R}}}^\mathrm{{lab}}$ (keV)	$\varGamma$ (eV)
632.23(4)	6.7(5)
991.86(3)	70(14)
1213.08(6)	<100
1587.49(8)	<200
1799.75(9)	450(60)

The second objective of this study was to check the long-term stability and energy reproducibility of the accelerated beam, another essential requirement of protracted data-taking experiments typical of low-energy nuclear astrophysics studies. For this purpose, multiple runs were taken at a fixed effective energy (i.e. accelerator settings) corresponding to the $E_{\mathrm{{R}}}^\mathrm{{lab}}$ = 992 keV resonance in the ^27^Al(*p*,$\gamma )^{28}$Si reaction, and their yields plotted as a function of time.

In the following sections we describe the experimental setup and procedures for data analysis (Sects. 2, 3), with results and conclusions presented in Sects. 5,  6, respectively.

**Fig. 2:**
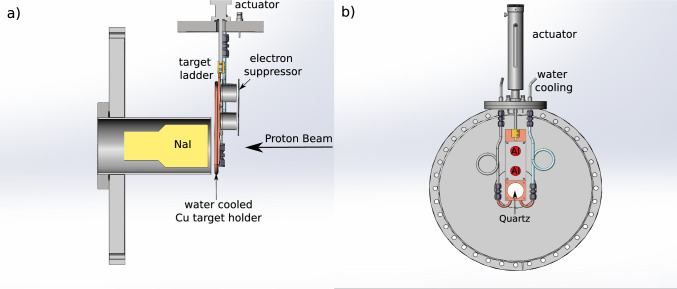
On the left (**a**) is the schematic representation of the set up used at DCF. The beam would pass through the electron suppressor and the Faraday cup to reach the Al target. The target was electrically isolated from the Cu plate that was used for heat dissipation. The NaI detector was placed behind the target bracket. On the right (**b**) is a head on view from the direction of the incident beam. One of the target positions was replaced with a quartz crystal to help with incident beam alignment

## Experimental setup

### Accelerator

The experiment was conducted using the 2.5 MV Pelletron accelerator (NEC Model 7.5SH-2). Equipped with a positive radio-frequency (RF) plasma source [13] in its terminal, the accelerator can generate ion beams – protons, helium, and heavier noble gases – up to a maximum current of 100 $\mu$A [7]. NEC Pelletrons are electrostatic accelerators and operate by using charging chains, driven from the ground end, to maintain a high-voltage charge on a terminal shell. Voltage stability is achieved through a terminal potential stabilizer (TPS, model 6.0) which adjusts the terminal charge through the regulation of a coronal discharge system to maintain the preset voltage. The TPS derives the terminal potential from a spinning vane-generating voltmeter (GVM) and two capacitive pickoffs (CPOs) contained in the walls of the pressure vessel. A corona probe is provided that the TPS utilizes to draw off excess charge from the terminal in order to maintain the terminal potential to the predefined voltage. Each TPS is calibrated specifically for its designated accelerator in the factory, with field adjustments discouraged by the manufacturer.

The general purpose irradiation chamber on Line A (see Fig. 1), the only one available to users for the 2.5 MV accelerator, has a $4^{\circ }$ deflection at the dipole switch magnet. This relatively small energy dispersion provides minimal energy filtering, allowing the experiment to test energy stability directly. A magnetic quadrupole near the accelerator’s exit refocuses the beam towards the beamline, while a second quadrupole just before the experimental end-station focuses the beam on the target. This section includes a Faraday cup (NEC model FC18) for beam current reading and a spinning wire beam position monitor (NEC model BPM80) for beam profile information. After an initial setup using the BPM80, the quadrupole focus was adjusted to project the beam focal point downstream onto a quartz slide mounted on the target ladder.^1^ Magnetic steerers at the end of the beamline ensured a precise alignment with the target.

**Fig. 3:**
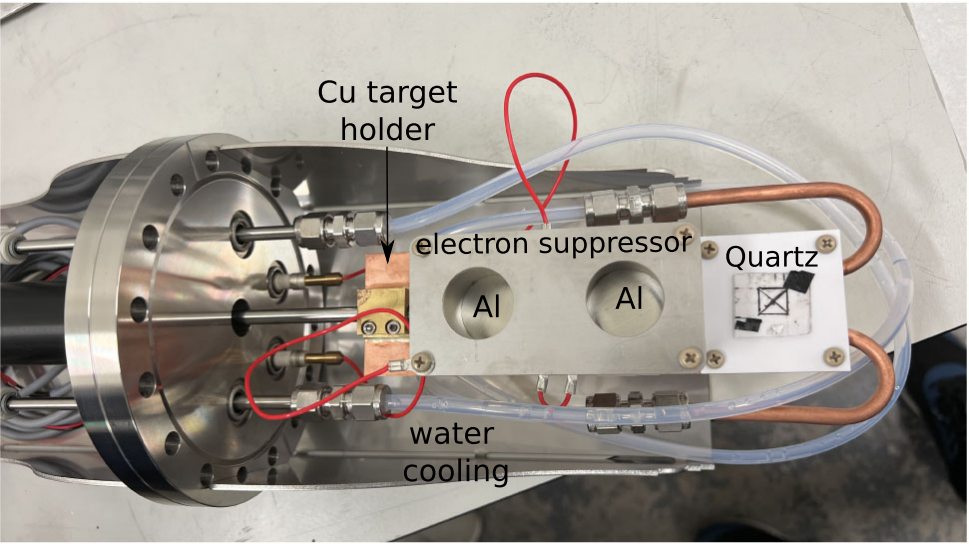
Close up view of the target ladder used to hold the two Al targets and a quartz crystal. Cu back plate and water cooling are used to dissipate heat

### Targets

Targets were produced at the Nuclear Science Laboratory of the University of Notre Dame (USA) [14] by evaporating ^27^Al onto 0.25-mm thick Ta backings. The backings were cleaned with acetone, ethyl alcohol, and semiconductor grade isopropyl alcohol, with ultrasonication applied at each step, finally followed by drying in an Ar flow. The Ta backings were then vacuum anneale ($P = 1.33 \times 10^{-6}$ mbar) using direct current heating to remove surface contamination. Aluminium metal (99.5% purity) was subsequently evaporated at a base pressure of $6.67 \times 10^{-6}$ mbar onto the Ta backings placed at 15 cm from the Al source boat to ensure uniform deposition. Real-time monitoring of the deposition was performed using Au-coated quartz crystals, with thickness measurements recorded by a monitor. The evaporation rate was maintained at $1.0~\mu$g/min to achieve $40-50~\mu$g/$\hbox {cm}^2$ areal density. At the end of the evaporation process, the targets were allowed to cool for 30 min before flushing the evaporator with high-purity argon gas.

For the measurements at DCF, two Al targets were mounted (50 mm apart) onto a water-cooled Cu plate attached to a target ladder as shown in Figs. 2 and 3. For beam current reading, the targets were surrounded by metallic cylinders, acting as small Faraday cups, and secured to the Cu plate with polyether ether ketone (PEEK) screws. Electrical insulation between the Al targets and the Cu plate was achieved by placing a 0.5 mm aluminum nitride wafer underneath the targets. A metallic plate with circular apertures of diameter slightly smaller than that of the cylinders was mounted above the cylinders (but not in contact) and maintained at a constant negative potential (typically – 300 V) with respect to the target for electron suppression, thus ensuring a correct beam current reading during the measurements (see Sec. 3 for details). PEEK standoffs were used to hold the electron suppression plate in position in front of the cylinders. All electrical connections were made with polytetrafluoroethylene (PTFE) insulated wire. Finally, a 0.5 mm Macor ceramic sheet was placed in the lowest position of the target ladder (see Fig. 2, panel b)), with a small piece of fused quartz affixed onto it with carbon tape. The quartz was used to verify beam alignment. Positioning of either of the Al targets and of the quartz piece was achieved by a mechanical actuator that allowed a vertical displacement of up to 100 mm to cover the full range of the target ladder. The actuator was mounted on a CF16 flange, with water and electrical feedthroughs, and fitted with an RS485 motor controller for remote operation via a local computer. Both the Al targets and the electron suppression plate were biased using an Iseg NHS 60 30p High Voltage Power Supply, with values remotely set, controlled, and stored using Python scripts.

### Detector and electronics

The $\gamma$ rays produced by the ^27^Al(p,$\gamma )^{28}$Si reaction were detected using a $3''\times 3''$ sodium iodide NaI(Tl) (Saint-Gobain 3M3/3-X [15]) detector placed at $0^{\circ }$ with respect to the beam axis and housed in a re-entrant CF100 flange mounted on the target DCF chamber. The detector was operated at a bias of +700 V supplied by a Brandenburg 477 power supply. The output signal was first pre-amplified using an ORTEC 113 scintillation preamplifier and then further amplified with an ORTEC 572 amplifier. The amplified signal was sent to an Ortec Easy MCA 2k, operated by the Maestro Multichannel Analyzer (MCA) Emulation Software, and the data were saved in ASCII format for offline analysis. The detector energy calibration was performed using common natural background radiation peaks accumulated during an overnight run without beam on target. A calibrated spectrum can be found in the Supplemental Material [16]. The observed energy resolution was $\varDelta E_{\gamma } \mathrm{(FWHM)} = 80$ keV at $E_{\gamma } = 661$ keV, i.e. 12%.

**Fig. 4:**
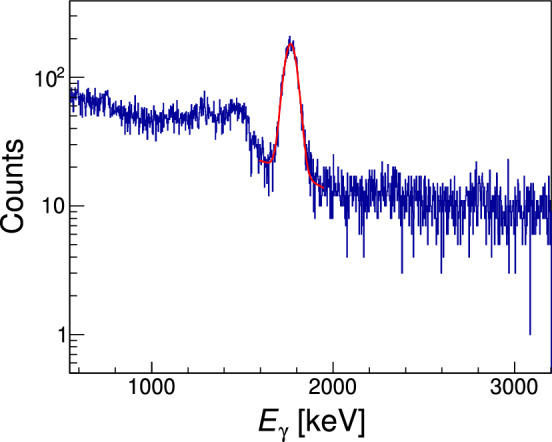
Typical $\gamma$-ray spectrum from one of the runs of the $E_{\mathrm{{R}}}^\mathrm{{lab}}$ = 992 keV resonance scan. The fit to the 1779-keV $\gamma$-ray peak using Eq. 1 is shown in red

## Experimental procedure

The 2.5 MV Pelletron accelerator delivered proton beams with intensities up to 3 $\mu$A. The terminal voltage (TV) and extraction voltage (EV) of the accelerator were initially set to values such that the nominal beam energy (TV + EV) was about 1–2 kV above a given resonance energy $E_{\mathrm{{R}}}^\mathrm{{lab}}$.

To perform a full resonance scan for each of the resonances studied (Table 1), we then progressively changed the beam energy by applying a positive bias voltage to the target in steps of 100–300 V up to 3 kV, while also maintaining a relative negative potential (typically, 200–300 V) between the target and the electron suppression plate. Runs were acquired for a fixed time at each step.

To check the long-term stability of the accelerator, we also monitored changes in the reaction yield as a function of time, over a period of about 70 min. To this end, we performed several 2 min runs with machine and target bias parameters fixed at an effective voltage close to the $E_{\textrm{R}}^\textrm{lab} = 992$ keV resonance. More details on the analysis techniques and results are discussed in Sects. 4 and 5.

## Data analysis

**Fig. 5:**
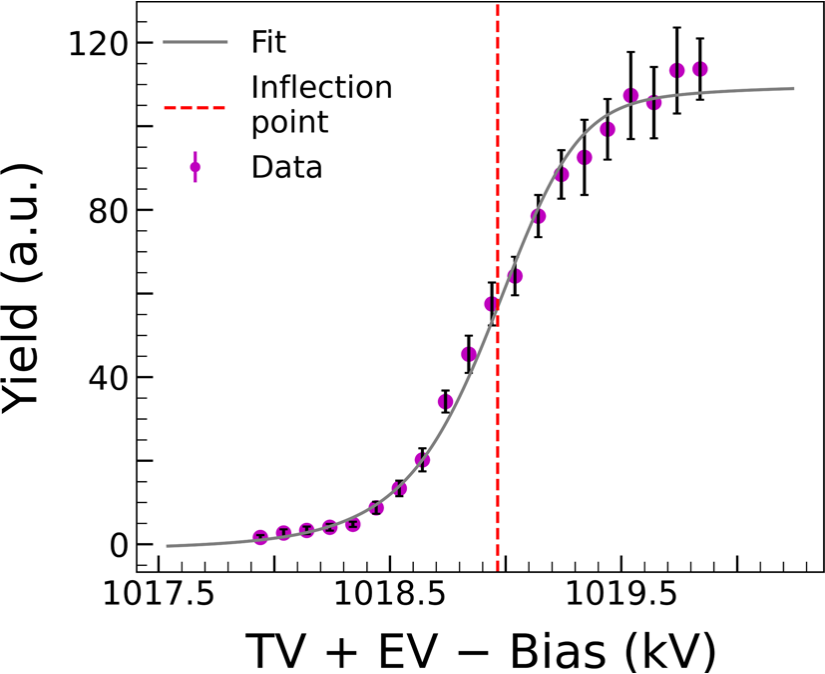
Thick target yield curve for the 992 keV resonance measurement. The gray line represents the fitting curve calculated using the Eq. 6. The red dashed line is the inflection point of the curve which is related to the resonance energy in the laboratory frame $E_{\mathrm{{R}}}^\mathrm{{lab}}$

All resonances exploited in this study de-excite via successive $\gamma$-ray transitions through the first excited state ($E_x = 1779.030(11)$ keV) of ^28^Si, ending with the emission of a secondary $\gamma$ ray with energy $E_{\gamma } = 1778.969(11)$ keV [17]. For the purpose of our analysis, we therefore selected a region of interest around this $\gamma$-ray peak and performed a Gaussian fit with an added linear background to determine the net counts *N* under the peak, as:

1 $$\begin{aligned} f(x) = N\frac{1}{\sigma \sqrt{2\pi }} \textrm{exp}\left( \frac{-(x-\mu )^2}{2\sigma ^2}\right) + ax + b, \end{aligned}$$

where $\sigma$ is the standard deviation, $\mu$ is the mean, and *a* and *b* are coefficients of the first order polynomial fit accounting for the background. An example of the fit is shown in Fig. 4. The yield *Y* was then obtained at each nominal beam energy as:

2 $$\begin{aligned} Y = \frac{N}{Q}, \end{aligned}$$

where *Q* is the total accumulated charge from the incident proton beam in a given measurement run. A plot of *Y* vs (TV + EV − Bias) finally gives the thick-target yield for each resonance. This is characterized by an initial flat plateau, followed by a steep rise and leveling off at the maximum yield. An example of the 992 keV scan is shown in Fig. 5. Yield curves for all other resonances are reported in the Supplemental Material [16].

In order to accurately determine the inflection point of the rising edge and to determine the energy spread of the beam as it comes from the accelerator, a fit to the data points of the resonance scan plot is required that takes into account all the physical effects influencing the shape of the yield curve.

For a narrow resonance at an energy $E_{\mathrm{{R}}}$ and with width $\varGamma$, the yield *Y* can be expressed as [18, 19]:

3 $$\begin{aligned} Y(E_p)&= \int _{E_p - \varDelta E} ^{E_p} \frac{\sigma (E)}{\epsilon (E)} \textrm{d}E \nonumber \\&= { k} \int _{E_p - \varDelta E} ^{E_p} \sigma _{\textrm{BW}} (E, E_{\textrm{R}}, \varGamma ) \textrm{d}E , \end{aligned}$$

where $E_{p}$ is the proton beam energy, $\varDelta E$ is the target thickness (expressed as energy lost by the beam in traversing the target), $\sigma _{\textrm{BW}} (E)$ is the Breit-Wigner cross section of the resonance, and $\epsilon (E)$ is the effective stopping power (here assumed constant over the energy $\varDelta E$ and taken out of the integral as *k*).

**Fig. 6:**
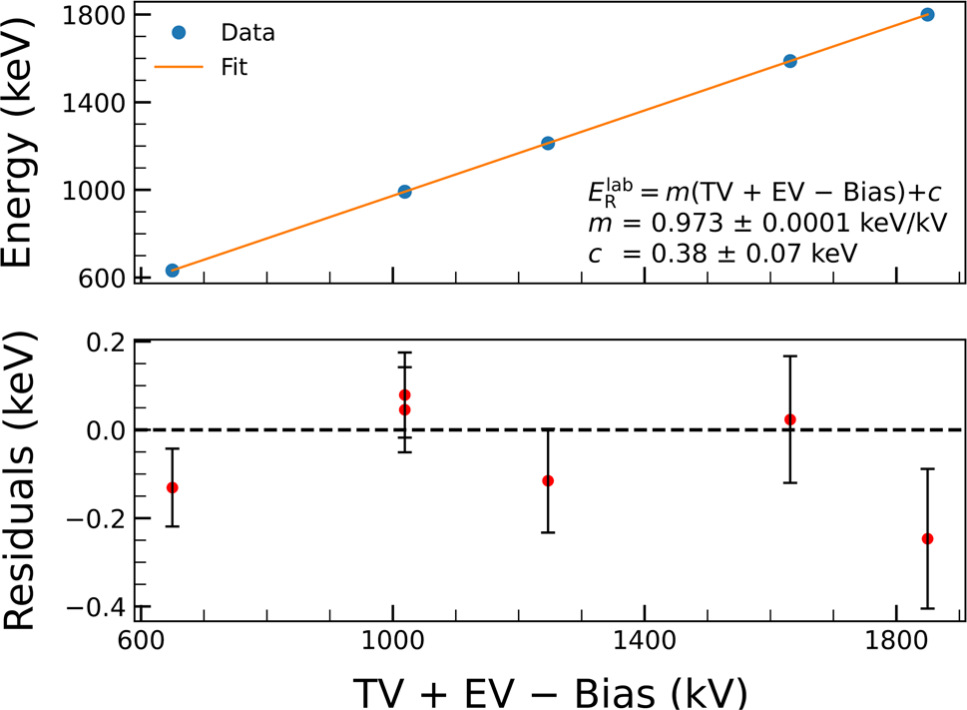
Energy calibration plot of the 2.5 MV accelerator as obtained from five well-known resonances in ^27^Al($p,\gamma )^{28}$Si reaction. The calibration coefficients *m* and *c* were obtained from a linear fit to the experimental data points. The residual plot is shown in the lower panel

**Fig. 7:**
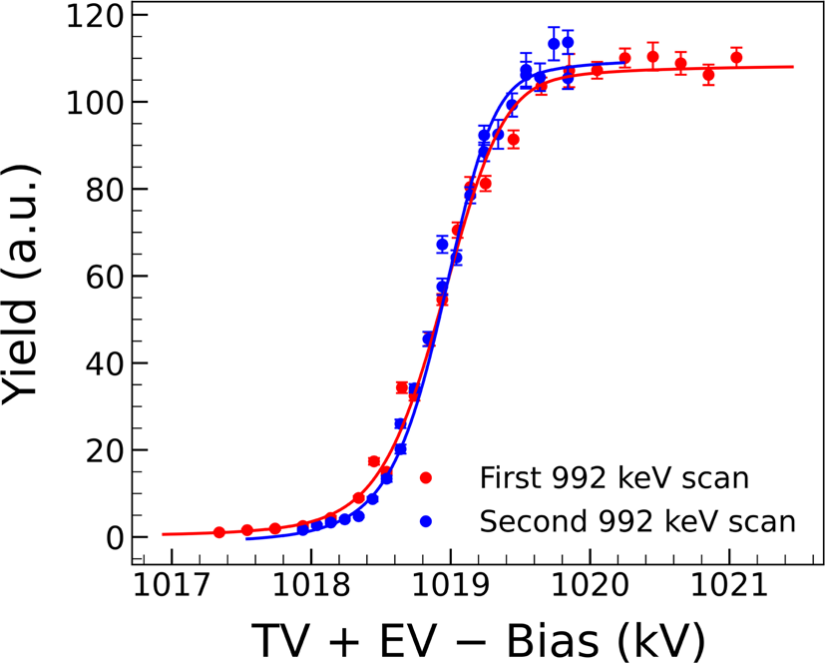
Comparison between two scans of the 992 keV resonance showing good reproducibility and similar beam energy spread (see text for details)

**Fig. 8:**
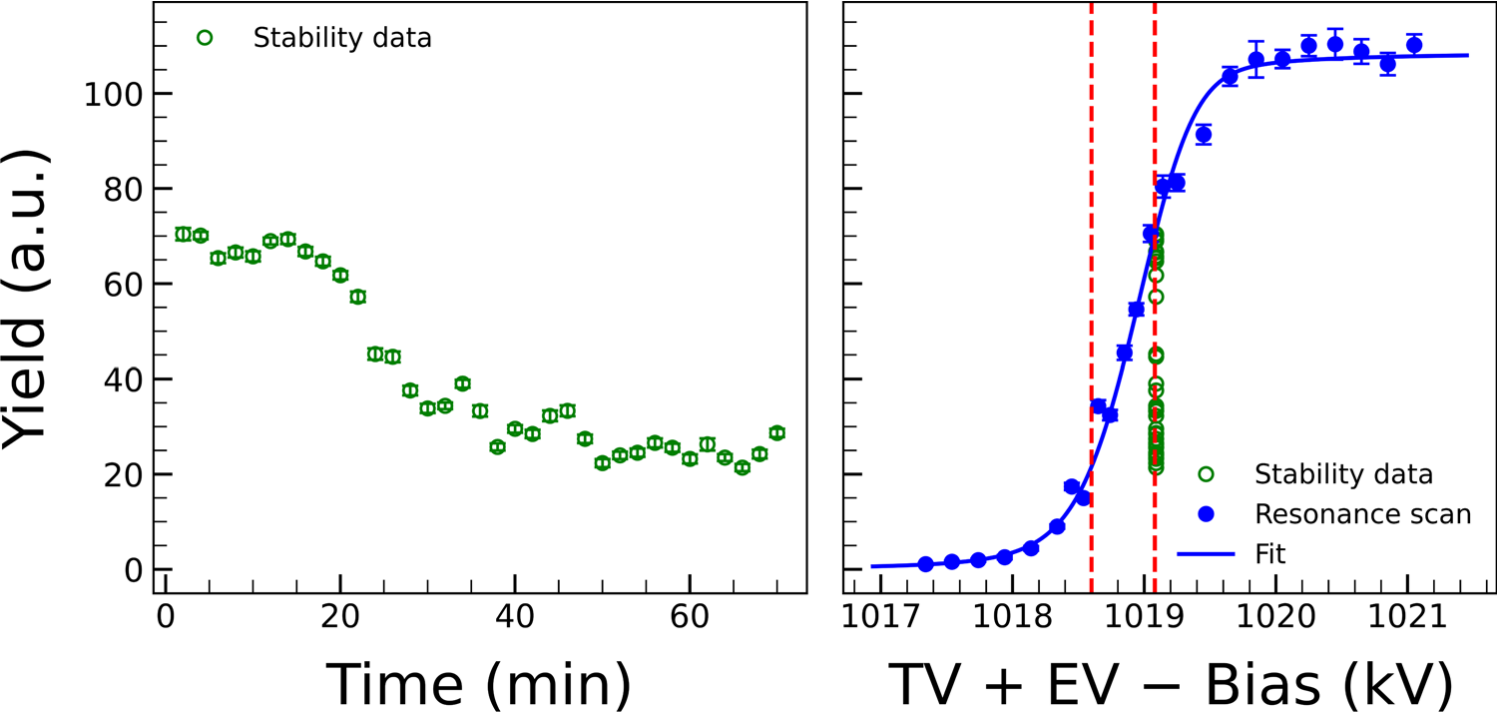
On the left, the yield obtained from various 2 min runs, all taken with the same voltage parameters (TV $+$ EV − Bias), is shown as a function of time. The voltage parameters for the measurement were set close to the resonance energy, so even a small change in voltage leads to a significant change in yield. By comparing the observed yield with the fit for the 992 keV resonance, the voltage drift was found to be 480 V. The voltage drift is indicated on the plot on the right as the region between the two red dashed lines

We note that the beam is not mono-energetic but characterized by a total energy spread given by:

4 $$\begin{aligned} \sigma _{\textrm{total}} = \sqrt{\sigma _{beam }^2 + \sigma _{straggling }^2 + \sigma _{Doppler }^2} \end{aligned}$$

Here, $\sigma _{beam }$ is the intrinsic energy spread (as characteristic of the accelerator); $\sigma _{{straggling} }$ is the spread associated to beam interactions in the target (see Ref. [20] for a derivation); and $\sigma _{{Doppler} }$ is the broadening arising from the thermal motion of the target atoms. This latter can be calculated as [21]:

5 $$\begin{aligned} \sigma _{Doppler } = \sqrt{\frac{2m_{p} E_p k_B T}{m_{\textrm{t}}}}, \end{aligned}$$

where $m_p$ and $m_{\textrm{t}}$ are the masses of the projectile and target, respectively, $k_B$ is the Boltzmann constant, and *T* is the target surface temperature (assumed to be ca. $80^{\circ }$C, based on previous experience for similar setups and experimental conditions). Typical values obtained here were in the range 40–60 eV.

In order to properly describe the observed yield, Eq. 3 must then be convoluted with a Gaussian function $F(E, E_{i}, \sigma _{\textrm{total}})$ as:

6 $$\begin{aligned} Y = { k} \int \textrm{d}E_{i} \int _{E_p - \varDelta E} ^{E_p} F(E_{i},E,\sigma _{\textrm{total}}) \sigma _{\textrm{BW}}(E, E_{R }, \varGamma ) \textrm{d}E. \end{aligned}$$

In the fit, *k*, $\sigma _{beam }$ and $E_{R }$ were left free to vary, and $\sigma _{straggling }$ was calculated using the method reported in Ref. [20]. Specifically, from a fit to the best-known 992 keV resonance (Fig. 5) and Eq. 4, the beam spread was calculated to be $\sigma _{\textrm{beam}} = 191(38)$ eV. Similar values were obtained from the fitted yields of other resonances whose $E_{\textrm{R}}$ and $\varGamma$ values are well known.

To arrive at the energy calibration of the 2.5 MV accelerator, resonance scans were repeated for all five energies in $E_{\mathrm{{R}}}^\mathrm{{lab}}$ = 632–1800 keV range, and their energies correlated to the effective voltage parameters (TV + EV − Bias) of the inflection point, at 50% of maximum yield from the best-fit curve.

## Results

### Accelerator’s energy calibration parameters

In total, six measurements for five resonance energies have been performed for the energy calibration of the accelerator. For the 992 keV resonance, the measurement was done twice. Data points are shown in Fig. 6 as a function of accelerator voltage parameters and target bias voltage. The best fit obtained with a linear function:

7 $$\begin{aligned} E_{\mathrm{{R}}}^\mathrm{{lab}} = m (\mathrm {TV + EV - Bias}) + c, \end{aligned}$$

leads to energy calibration parameters $m =0.9730(1)$ keV/kV and $c = 0.38(7)$ keV, with residuals shown in the bottom panel of Fig. 6. The uncertainties on the residuals correspond to the uncertainties on the resonance energies taken from Ref. [12]. It should be noted that EV and Bias are independently controlled and precisely monitored by commercial high-voltage power suppliers (for the measurements reported here, EV was maintained at 16.4 kV to within 10V). However, for the purpose of our analysis, we opted to treat the system as a whole, i.e., using a single calibration coefficient *m*, especially given the dominant influence of the TV term.

### Reproducibility and stability checks

To check the beam-energy reproducibility, two 992 keV resonance scans were performed on different days, and shown in Fig. 7. The difference in $\sigma _{\textrm{beam}}$ between the two measurements was found to be 42 eV, which is within 1.1$\sigma$ of $\sigma _{\textrm{beam}}$ = 191(38) eV value.

To further check on the beam-energy long-term stability, we took several 2 min yield measurements over a period of 70 min (because of the limited beam time available, it was not possible to extend such measurements over longer times). All runs were taken at a fixed effective voltage (TV + EV − Bias) corresponding to the 992 keV resonance. Results, shown in Fig. 8 (left panel), indicate a gradual decrease in yield with respect to time, possibly due to a small drift of the accelerator voltage settings. To determine the magnitude of such a drift, we display the 2 min yield values alongside the 992 keV resonance scan fit as shown in Fig. 8 (right panel). The previous fit of the 992 keV resonance scan allowed us to infer what accelerator settings (TV $+$ EV − Bias) would match the measured yields during the stability runs. The maximum voltage drift of the accelerator can then be determined from the difference between accelerator settings corresponding to the maximum and minimum yields observed. This difference is indicated by the two dashed lines in Fig. 8 (right panel) and corresponds to a value of 480 V. More data would be required to find any systematic trend in the stability behavior of the beam for future experiments.

## Conclusions

We have used five well-known resonances in the ^27^Al($p,\gamma )^{28}$Si reaction to successfully perform the first beam-energy calibration measurement of the 2.5 MV Pelletron at the Dalton Cumbrian Facility in England, UK. The energy calibration parameters are *m* = 0.9730(1) keV/kV and *c* = 0.38(7) keV, with a typical beam spread $\sigma _{\textrm{beam}} = 191(38)$ eV, as obtained from a fit to the thick-target yield of the $E_{\mathrm{{R}}}^\mathrm{{lab}} = 992$ keV resonance. We further checked the beam energy reproducibility for two of the investigated resonances, and investigated the long-term energy stability of the beam over a period of $\sim$ 70 min. While yield values indicate a small voltage drift of 480 V, more data would be desired to make conclusion on the suitability of the Dalton Cumbrian Facility for nuclear astrophysics reaction studies at sub-Coulomb energies, where long-term stability is needed. Nevertheless, the accelerator is well-suited for measurements at higher energies, and provides a useful complement to other surface and underground laboratories (such as LUNA [2], CASPAR [22], Felsenkeller [23], JUNA [24]) for the study of reactions with stable beams in hydrogen-, carbon-, and oxygen-burning under high-temperature conditions. With its high energy resolution, the Dalton facility offers the nuclear astrophysics community in the UK an excellent opportunity to advance research in this field.

## Supplementary Information

Below is the link to the electronic supplementary material.

Supplementary file 1 (pdf 1866 KB)

## Data Availability

Data will be made available on reasonable request. [Author’s comment: The datasets generated during and/or analysed during the current study are available from the corresponding author on reasonable request.]

## Footnotes

1 When struck by a beam of charged particles, the quartz emits visible light which can be viewed with a camera to aid the beam focusing procedure.

## Code Availability

Code/software will be made available on reasonable request. [Author’s comment: The code/software generated during and/or analysed during the current study is available from the corresponding author on reasonable request.]
