# Supplementary material for: Energy calibration of the 2.5 MV Pelletron at the Dalton Cumbrian Facility
Source: Eur Phys J A Hadron Nucl. 2025 Jul 8;61(7):155. doi: 10.1140/epja/s10050-025-01622-5 (PMC12238158; doi:10.1140/epja/s10050-025-01622-5)
Supplement: Supplementary file 1 — (pdf 1866 KB) [file 10050_2025_1622_MOESM1_ESM.pdf]

EPJ manuscript No.
(will be inserted by the editor)

Supplemental Material for: Energy Calibration of the 2.5 MV Pelletron at the Dalton Cumbrian Facility

K. Linkowski[1], R. S. Sidhu[1,2a], J. Skowronski[3,4], M. Aliotta[1b], P. Black[1], T. Davinson[1], M. Wiescher[5,1], A. Caciolli[3], J. Jones[1], K. Manukyan[5], D. Robertson[5], and A. Smith[6]

[1] School of Physics and Astronomy, The University of Edinburgh, EH9 3FD Edinburgh, UK
[2] School of Mathematics and Physics, University of Surrey, Guildford, GU2 7XH, UK
[3] Università degli Studi di Padova, 35131 Padova, Italy
[4] INFN, Sezione di Padova, 35131 Padova, Italy
[5] Department of Physics and Astronomy, University of Notre Dame, Notre Dame, Indiana 46556, USA
[6] The University of Manchester's Dalton Cumbrian Facility, Westlakes Science Park, Moor Row, Cumbria CA24 3HA, UK

July 16, 2025

Abstract. We report on the energy calibration of the 2.5 MV Pelletron accelerator at the Dalton Cumbrian Facility in England (UK) using five well-known resonances in the ^{27}Al(p,γ)^{28}Si reaction in the proton beam energy range of $632 - 1800$ keV. The beam energy spread was also measured and found to be 191(38) eV. Additionally, we checked the stability and reproducibility of the accelerator's beam energy, confirming its suitability for nuclear astrophysics experiments, especially in the high-energy regime of stellar evolution and nucleosynthesis.

Key words. Low-energy nuclear astrophysics, Particle accelerators, Energy calibration, Nuclear reactions

PACS. PACS-key discribing text of that key – PACS-key discribing text of that key

1 Calibration of the detector

The energy calibration of the NaI detector was performed using γ-ray peaks from an overnight-long natural background run taken without beam on target. The background spectrum is shown in Fig. 1.

2 Thick-target yield plots

Thick-target yield plots are shown in Figs. 2, 3, 4, 5, and 6, for all five resonances used in this study, respectively at E_{p} = 632 keV, 992 keV, 1213 keV, 1587 keV, and 1800 keV.

[a] email: ragan.sidhu@surrey.ac.uk (corresponding author)
[b] email: m.aliotta@ed.ac.uk (corresponding author)

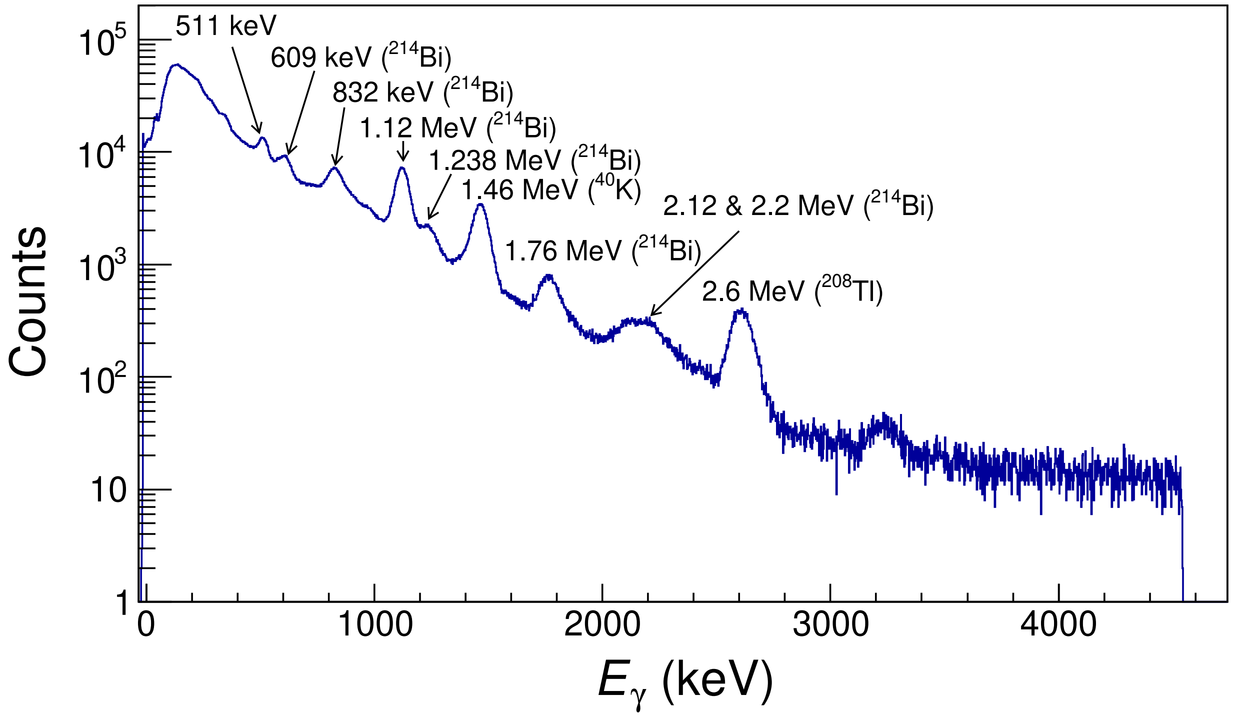

Fig. 1. Natural background spectrum obtained in an overnight run with the 3″ × 3″ NaI detectors.

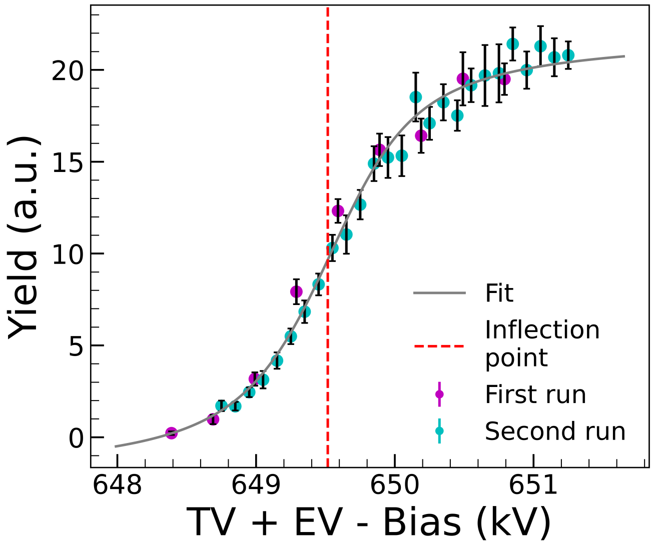

Fig. 2. Thick-target yield curve for the E_p = 632 keV resonance scan. The two scans were taken in rapid succession, one after the other.

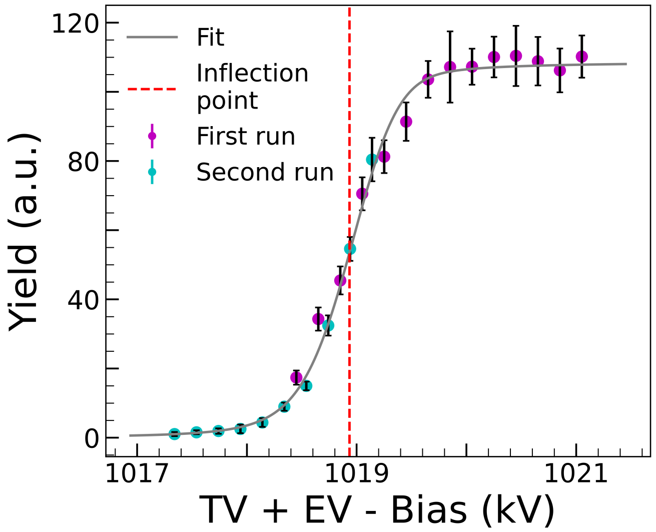

Fig. 3. Thick-target yield curve for the E_p = 992 keV resonance scan. The two scans were taken about one day apart from each other.

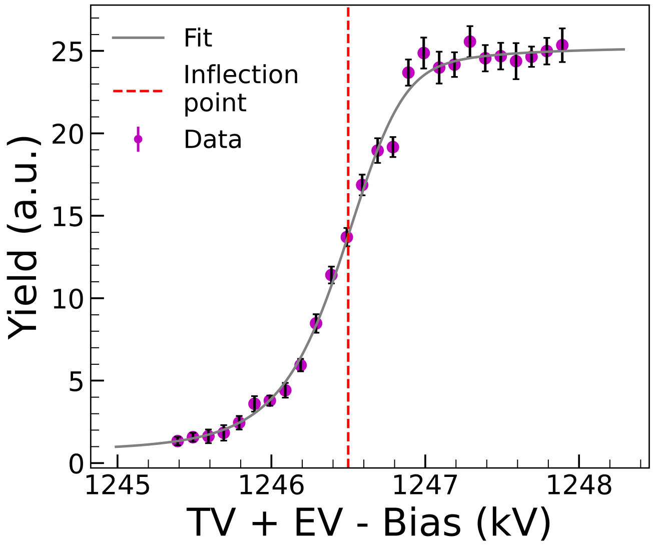

Fig. 4. Thick-target yield curve for the E_p = 1213 keV resonance scan.

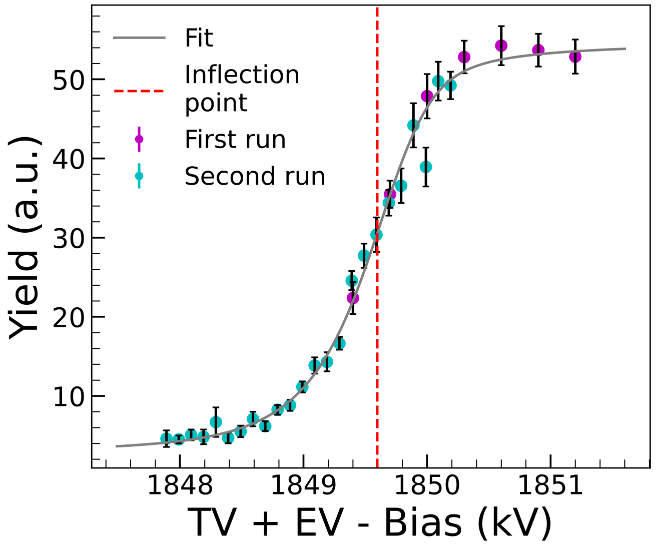

Fig. 6. Thick-target yield curve for the E_p = 1800 keV resonance scan.

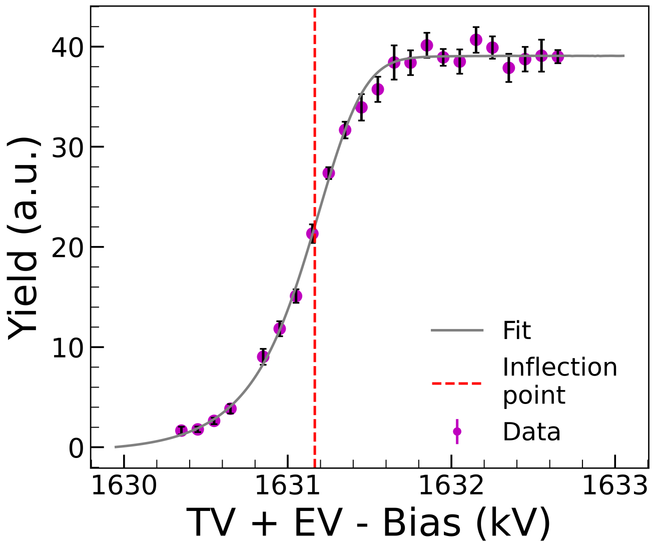

Fig. 5. Thick-target yield curve for the E_p = 1587 keV resonance scan.
